# Nupr1-mediated vascular smooth muscle cell phenotype transformation involved in methamphetamine induces pulmonary hypertension

**DOI:** 10.1007/s10565-024-09849-6

**Published:** 2024-02-13

**Authors:** Jie Zhou, Dan Guo, Zhen-Zhen Xu, Jia-Shun Liao, Xiao-Ting Li, Ke Duan, Shi-You Chen, Wei-Bing Xie

**Affiliations:** 1 Guangzhou Key Laboratory of Forensic Multi-Omics for Precision Identification, School of Forensic Medicine, Southern Medical University, Guangzhou, 510515 People’s Republic of China; 2 Department of Pharmacy, Nanfang Hospital, Southern Medical University, Guangzhou, 510515 People’s Republic of China; 3 Department of Surgery, Medical Pharmacology & Physiology, University of Missouri, Columbia, MO 65212 USA

**Keywords:** Methamphetamine, Pulmonary hypertension, Nupr1, Phenotype transformation, Vascular smooth muscle cell

## Abstract

**Aims:**

Nuclear protein 1 (Nupr1) is a multifunctional stress-induced protein involved in the regulation of tumorigenesis, apoptosis, and autophagy. However, its role in pulmonary hypertension (PH) after METH exposure remains unexplored. In this study, we aimed to investigate whether METH can induce PH and describe the role and mechanism of Nupr1 in the development of PH.

**Methods and results:**

Mice were made to induce pulmonary hypertension (PH) upon chronic intermittent treatment with METH. Their right ventricular systolic pressure (RVSP) was measured to assess pulmonary artery pressure. Pulmonary artery morphometry was determined by H&E staining and Masson staining. Nupr1 expression and function were detected in human lungs, mice lungs exposed to METH, and cultured pulmonary arterial smooth muscle cells (PASMCs) with METH treatment. Our results showed that chronic intermittent METH treatment successfully induced PH in mice. Nupr1 expression was increased in the cultured PASMCs, pulmonary arterial media from METH-exposed mice, and METH-ingested human specimens compared with control. Elevated Nupr1 expression promoted PASMC phenotype change from contractile to synthetic, which triggered pulmonary artery remodeling and resulted in PH formation. Mechanistically, Nupr1 mediated the opening of store-operated calcium entry (SOCE) by activating the expression of STIM1, thereby promoting Ca^2+^ influx and inducing phenotypic conversion of PASMCs.

**Conclusions:**

Nupr1 activation could promote Ca^2+^ influx through STIM1-mediated SOCE opening, which promoted METH-induced pulmonary artery remodeling and led to PH formation. These results suggested that Nupr1 played an important role in METH-induced PH and might be a potential target for METH-related PH therapy.

**Graphical Abstract:**

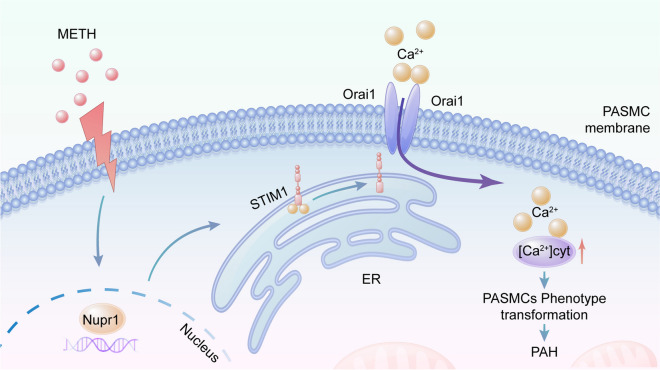

1. Chronic intermittent methamphetamine exposure can induce the development of pulmonary arterial hypertension.

2. Nupr1 plays a key role in the development of methamphetamine-related pulmonary arterial hypertension.

3. Nupr1 mediates PASMCs phenotypic transformation via STIM1signal axis, which results in the development of methamphetamine-related pulmonary arterial hypertension.

**Supplementary Information:**

The online version contains supplementary material available at 10.1007/s10565-024-09849-6.

## Introduction

Pulmonary hypertension (PH) is a highly malignant progressive disease characterized by a gradual increase in pulmonary arterial resistance, leading to increased right heart afterload and eventual death from right heart failure. Its main pathological features include the vasospasm of pulmonary arterioles, hyperplasia of pulmonary intima, and vascular remodeling (Spiekerkoetter et al. 2019). Recently, more and more data have shown that the frequency and risk of PH are significantly increased in chronic methamphetamine (METH) abusers (Orcholski et al. 2017; Kolaitis et al. 2021; Kolaitis and Saggar 2022; Prohaska and Machado 2021).

METH abuse damages the human body’s main organs and induces related diseases. The lung is the main organ for the absorption and accumulation of METH after entering the human body, accounting for 24–31% of the total intake (Volkow et al. 2010). Locally high concentrations of METH have serious toxic damage to lung tissues, causing lung-related diseases, among which PH has attracted researchers’ attention due to its hidden course, poor prognosis, and disastrous consequences. It has now been confirmed that METH can cause a rapid increase in the heart rate and blood pressure of smokers after entering the human body (Carracedo et al. 2006), mostly related to METH’s ability to induce the synthesis and release of catecholamines. However, despite numerous reports on PH describing the effects of long-term abuse of METH (Cai et al. 2016; Xu et al. 2017), there is limited literature on the occurrence and effects of systemic hypertension in METH abusers, which we believe require increasing focus due to the underlying poor prognosis and high mortality of the sufferers. Although there is a consensus on the occurrence of METH-PH (methamphetamine-related pulmonary hypertension), the mechanism of METH-PH remains far from being clarified. Thus, deeper investigations on this topic could provide a theoretical basis to formulate strategies for preventing and treating METH-PH.

A primary contributor to the onset of PH is increased pulmonary vascular resistance due to pathological changes such as persistent vasoconstriction and excessive pulmonary vascular remodeling (Hoeper et al. 2013; Luna-Lopez et al. 2022; Naeije et al. 2022). The proliferation of pulmonary artery smooth muscle cells (PASMCs) is the basis of this pathological change. In the physiological state, PASMCs have contractile functions and maintain the pressure of blood vessels, while in the pathological state, phenotypic transformation occurs and turns into a proliferative type, whereby smooth muscle cells begin to proliferate, leading to vascular remodeling (Rabinovitch 2012). Elevated cytoplasmic Ca^2+^ concentration ([Ca^2+^]_cyt_) in PASMCs serves as a pivotal initiator for pulmonary vasoconstriction, representing a significant inducer of pulmonary vascular remodeling (Kuhr et al. 2012).

The endoplasmic reticulum (ER) stands as a crucial organelle within eukaryotic cells (Díaz-Villanueva et al. 2015). Cells, when subjected to some internal or external factors, produce endoplasmic reticulum stress to respond to the stimulus (Schröder 2008). Previous studies have shown that the role of Nupr1 is upstream of ER stress response in the tetracycline-induced apoptosis of mouse and human tumor cells (Carracedo et al. 2006). Recent studies in our laboratory have also shown that the role of Nupr1 is upstream of the ER stress response in the autophagy and apoptosis of nerve cells (Xu et al. 2017) and vascular endothelial cells (Cai et al. 2016) induced by METH. In addition, the ER acts as a major intracellular Ca^2+^ storage compartment, which is crucial for maintaining Ca^2+^ homeostasis in various organelles (Kaufman et al. 2002; Krebs et al. 2015). Stromal interaction molecule 1 (STIM1), calcium release-activated calcium modulator 1 (Cracm1, also known as Orai1), and transient receptor potential canonical 1 (TRPC1) are considered the molecular components of store-operated Ca^2+^ channels (SOCCs) (Vig et al. 2006; Yeromin et al. 2006). STIM1 is located in ER membranes and is an endoplasmic reticulum Ca^2+^ level sensor that mediates SOCE. Store-operated calcium entry (SOCE) is activated by ER Ca^2+^ depletion and is the primary mechanism for the regulation of Ca^2+^ inward flow in nonexcitable cells (Yeromin et al. 2006; Zhu et al. 2021). And it has been demonstrated that Orai1 and TRPC play key roles in PH formation (Masson et al. 2023, 2022).

This study aimed to investigate whether METH causes PH and to elucidate the underlying mechanisms. Our results showed that METH could indeed induce PH in mice and that Nupr1 expression was upregulated in METH-PH animal models and METH users. Mechanistically, Nupr1 regulated the opening of the SOC calcium pathway, composed of STIM1/Orai1, by upregulating the expression of STIM1, leading to calcium influx and thus mediating the occurrence of METH-PH.

## Methods

### Animals and METH-PH model

Nupr1-knockout (Nupr1^−/−^) mice with a C57BL/6 background were generated through CRISPR/Cas9 gene editing under the auspices of the Animal Model Research Center at Nanjing University (Zhou et al. 2021). Six-week-old male C57BL/6 J mice were purchased from the Laboratory Animal Center of Southern Medical University (Guangzhou, China). Notably, considering the potential interference of estrogen-induced resistance to METH, exclusively male mice were employed in this study (Dluzen et al. 2002). The mice were housed in a controlled environment, maintaining a temperature of 22 ± 1 ℃, relative humidity of 50 ± 5%, and adhering to a 12-h light–dark cycle. They had ad libitum access to sterile food and water. Ethical considerations and animal welfare were prioritized, and all procedures were conducted in strict accordance with the NIH Guidelines for the Care and Use of Laboratory Animals (8th Edition, US National Research Council, 2011). The Institutional Animal Care and Use Committee of Southern Medical University approved the study protocol. The mice (*n* = 10 per group) were given METH at 10 mg/kg b.w. (> 99% purity [Guangzhou National Food and Drug Inspection Institute, China]), which was dissolved in 0.9% saline twice a day for 5 consecutive days, 2 days apart, a total of 10 weeks. Meanwhile, control mice were given saline using the same methods as METH. 50 mg/kg BrdU (B5002, Sigma-Aldrich) was intraperitoneally injected 3 days before the mice were sacrificed.

### Echocardiography

Mice were anesthetized by inhalation of isoflurane, and then a VisualSonics Vevo 2100 high-resolution ultrasound imaging system (USA) was utilized to capture and record images as a means of obtaining parameters such as pulmonary artery acceleration time (PAT), pulmonary artery ejection time (PET), and RV fractional area change (RVFAC).

### Right ventricular pressure detection

All surgical instruments were autoclaved. Mice were anesthetized by inhalation of isoflurane and kept breathing by a ventilator. After the toe contraction reaction disappeared, the mice were fixed on the platform. Then, a hair removal cream was used to remove the hair in the operation area, followed by skin disinfection using 75% alcohol. The skin, muscles, diaphragm, and ribs were cut with an ophthalmic scissor to expose the mice’s heart. Their right ventricular wall was penetrated with a syringe needle, the needle was taken out, and we gently pressed the puncture point with a cotton swab to prevent bleeding. Next, we punctured the ventricle with the tip of the catheter through the wound, and when the catheter tip was in the ventricle, the right ventricular pressure (RV) curve was displayed on the detector. When the curve was stable, we recorded the RV systolic pressure, RV diastolic pressure, RV dP/dt, and mouse heart rate until the systolic pressure continued to decrease. After the curve recording was completed, the catheter was pulled out and placed in normal saline.

### H&E and Masson staining

Lung tissues were processed as follows for histopathological examination. Initially, tissues were fixed in 4% paraformaldehyde for a duration of 48 h. Subsequently, the fixed tissues underwent a dehydration process using ethanol and were further embedded in paraffin. For histopathological analyses, 3-µm lung sections were stained with hematoxylin–eosin (H&E) or Masson staining reagents. The staining procedures were conducted using commercially available kits (Leagene Biotechnology) in accordance with the manufacturer’s protocol. The histological slides were examined and visualized using a Carl Zeiss microscope.

### Immunohistochemical staining

After 3-µm lung sections were deparaffinized in xylene as well as gradient ethanol hydration, antigen was repaired using citrate buffer. They were then treated with proteinase K for 10 min and 3% hydrogen peroxide for 10 min. The sections were washed three times with PBS between each step. Lung sections were then blocked in PBS containing 10% BSA for 1 h and incubated overnight at 4 °C with the following primary antibodies: anti-PCNA (ab29, Abcam, 20 µg/ml), anti-STIM1 (ab62031, Abcam, 20 µg/ml), anti-Nupr1 (bs7106R, Bioss, 20 µg/ml) at 4 ℃ overnight. The following day, after washing three times using PBS, the sections were incubated with biotinylated anti-rabbit/mouse secondary antibody for 10 min at room temperature. The sections were then stained with DAB (1:20) color solution in an environment protected from light. After staining with hematoxylin for 2 min, the slices were returned to blue in water for 30 min, and the slides were dehydrated and sealed for photography.

### Immunofluorescent staining

Here, we permeated the 3-µm lung sections or primary vascular smooth muscle cells with 0.1% Triton X-100 PBS solution for 15 min. Then, the lung sections were blocked with PBS containing 10% BSA for 1 h and incubated overnight with the following primary antibodies: anti-α-SMA (A2547, SIGMA, 20 µg/ml), anti-PCNA (ab29, Abcam, 20 µg/ml), anti-NUPR1 (bs7106R, Bioss, 20 µg/ml), anti-STIM1 (ab62031, Abcam, 20 µg/ml), anti-BrdU (ab6326, abcam, 1 µg/ml). Donkey anti-mouse IgG (A21202, Invitrogen, 4 µg/ml) and goat anti-rabbit IgG (A32740, Invitrogen, 4 µg/ml) were used as the secondary antibodies. DAPI (H-1200, Vector) was used for nuclear staining. After sealing the film, we took micrographs using an inverted laser confocal microscope (FV3000, Olympus).

### Pulmonary angiography and CT scanning

For pulmonary angiography, the mice were subjected to intraperitoneal injection of pentobarbital sodium (120 mg/kg) for anesthesia induction. A median sternotomy was conducted to open the chest, followed by the meticulous excision of the thymus and adipose tissue. A scalp needle was inserted into the right ventricle and immediately injected with heparin (20iu) to prevent blood clotting. The left atrial appendage was cut open, and the right ventricle was perfused with normal saline. After the lungs turned white, 4% paraformaldehyde was perfused. Then, the casting agent Microfil (MV-122, Flow Tech Inc.) was injected at 0.05 ml/min. Microfil composition consists of 600 µl of Microfil and 750 µl of diluent. Immediately preceding microfil perfusion, 67.5 µl of curing agent was added. The mice were left to stand for about 20 min and the Microfil solution solidified. The mice’s lungs were placed at 4 °C overnight and covered with wet paper to avoid dryness. The next day, the lungs were carefully separated and divided into two tests: ① the isolated lung tissues were placed in PBS and gently shaken at room temperature for 15 min. Dehydration of lung tissues was executed using gradients of ethanol (50%, 70%, 80%, 95%, and 100%) per hour to digest the lung tissues. Afterwards the lungs were immersed in methyl salicylate to visualize the pulmonary vasculature. The lungs were photographed with a Stereo Microscope (AxioZoomV16). ② The lung specimen was placed in a sample tube of 19-mm diameter, which was then wrapped with a plastic wrap three to four times to keep moisture. Foam was then placed on the top and bottom of the specimen to fix it and avoid artifacts caused by specimen movement during scanning. The sample was examined by Micro-CT (μCT100, Scanco Medical), during which the X-ray beam was scanned vertically with the sample surface. After scanning, the micro-CT system automatically performed a 2D reconstruction of the sample. Then, Scanco μCT Evaluation Program V6.6 software was used for data analysis and 3D reconstruction of the sample.

### Cell culture and drug treatment

Primary culture of mouse PASMCs was performed. Briefly, pulmonary arteries were first isolated from male C57BL/6 J mice, and their connective tissues were carefully removed under a light microscope. Following adventitia removal, the isolated pulmonary arteries were clipped and then digested in Dulbecco’s Modified Eagle’s medium (DMEM) containing 1 mg/ml type II collagenase (C8150, Solarbio) for 2 h at 37 °C. The digested PASMCs were centrifuged at 180 g for 5 min, then collected in culture bottles and subcultured with DMEM containing 10% fetal bovine serum (FBS) and antibiotics. All experiments only used PASMCs of early generation (three to eight generations). PASMCs were treated with METH (0–2.5 mM) for 36 h and then assayed by Western blot to observe the concentration-dependent effect of METH on the expression of proliferative- and contractile-related proteins. To explore the mechanism, PASMCs were treated with a Nupr1 inhibitor trifluoperazine (TFP) (T8516, Sigma). We pretreated PASMCs with 10 μM TFP for 30 min before treating them with 1.5 mM METH for 36 h.

### Cell proliferation

PASMCs was planted in confocal dishes and treated with METH (1.5 mM) 24 h later for 36 h, while the control group was treated with PBS for the same time. Staining was performed using an EDU Cell Proliferation Assay kit (C10310-1, RiboBio) according to the kit instructions.

### Small interfering RNA transfection

STIM1 siRNA#1 (sequence: 5′-GGTGGTATCTATCGTG ATT-3′), siRNA#2 (sequence: 5′-GTGATACAGTGGCTGATTA-3′), Nupr1 siRNA (sequence: 5′- CAAGTTCCAGAACTCTGAA-3′), and Orai1 siRNA (sequence: 5′-GCAACGCCACAACCUCAATT-3′) were designed and purchased from Ribobio (Guangzhou, China). According to the instructions of the transfection kit (RiboBio), cells were incubated with 100 nM siRNA in antibiotic-free DMEM containing 10% FBS at 37 °C, 5% CO_2_ in a humidified environment. After incubation for 24 h, they were treated with METH (1.5 mM) in DMEM containing 2% FBS for 24 h.

### Western blot

The protein samples were transferred to PVDF membranes after SDS-PAGE electrophoresis. After blocked with 5% skimmed milk, the membranes were incubated with specific primary antibodies at 4 °C overnight. The primary antibodies employed included anti-MMP2 (YT2798, Immunoway, 1 µg/ml), anti-MMP9 (10,375–2-AP, Proteintech, 1 µg/ml), or anti-β-actin (RM2001, Beijing Ray Antibody Biotech, 1 µg/ml). On the following day, PVDF membranes were washed and incubated with corresponding horseradish peroxidase HRP-labeled secondary antibody (Beijing Ray Antibody Biotech) for 1 h at room temperature. Finally, the membranes were developed with ECL chemiluminescence detection kit (Bio-Rad). After analyzing the grey values of the bands using Image J, β-actin was used as an internal reference for normalization and then statistically analyzed.

### Intracellular Ca^2+^ Measurement

The measurement of intracellular Ca^2+^ levels was conducted employing Fura-4 AM (S1060, Beyotime). Following the manufacturer’s guidelines, cells were subjected to three washes with a calcium-free HBSS solution before being loaded with 2 μM Fura-4 AM in HBSS for a 30-min incubation period. Subsequent to loading, cells underwent an additional three washes with HBSS and were incubated for an additional 20 min. Finally, 2 mM of Ca^2+^ was added and then the fluorescence intensity was detected by kinetic assay using a microplate reader.

### Human specimens

Human specimens were obtained from the Forensic Identification Center of Southern Medical University (Guangzhou, China). Specifically, METH specimens were collected from the deceased with a history of METH abuse, while control specimens were randomly collected from individuals who died of other causes. The ethical procedures for collecting these human specimens received approval from the Institutional Review Board of Southern Medical University.

### Statistical analysis

GraphPad Prism 8 was used for statistical analysis. Data were presented as mean ± standard deviation (SD). Two-group analyses were performed using the unpaired Student *t* test when the sample data obeyed a normal distribution and the Wilcoxon rank sum test when the sample data did not obey a normal distribution. Multiple groups of data were evaluated by one-way ANOVA or two-way ANOVA followed by Bonferroni multiple comparison test. A significance level of *P* < 0.05 was adopted to determine statistical significance. *P* < 0.05 was considered to be statistically significant.

## Results

### Chronic intermittent METH exposure induced PH

**Fig. 1:**
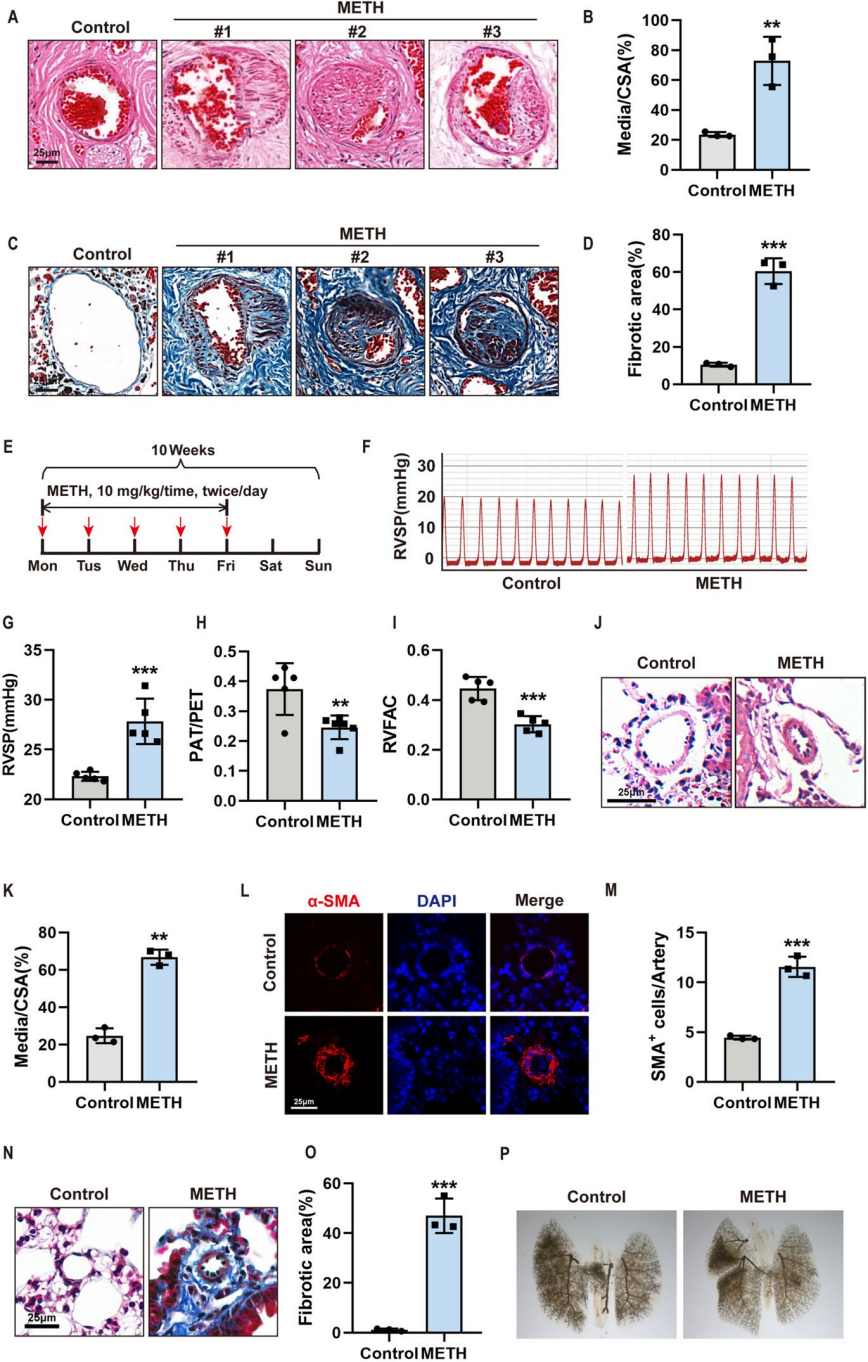
METH exposure induced PH formation. **A** Representative images of H&E-stained lung tissue sections of the deceased individuals with a METH abuse history. Scale bar, 25 μm. **B** Quantification ratio of vascular medial thickness to total vessel size in the pulmonary artery from the deceased individuals with METH abuse history and normal lungs (*n* = 3). Two-tailed unpaired *t* test. **C** Representative images of Masson-stained lung tissue sections of the deceased individuals with a METH abuse history. Scale bar, 25 μm. **D** Quantification of the fibrotic areas (%) by normalizing the Masson’s positive staining area to the total tissue area in the images analyzed in the pulmonary artery from the deceased individuals with a METH abuse history (*n* = 3). Two-tailed unpaired *t* test. **E** Schematic diagram showing the pulmonary hypertension mice model. **F** Pressure curve of right ventricular systolic pressure (RVSP) in mice exposed to METH. **G** Quantification of the right ventricular systolic pressure (RVSP) in mice exposed to METH (*n* = 5). Two-tailed unpaired *t* test. **H** Quantification of PAT/PET in mice exposed to METH (*n* = 5). Wilcoxon rank sum test. **I** Quantification of RVFAC in mice exposed to METH (*n* = 5). Two-tailed unpaired *t* test. **J** Representative images of H&E-stained lung tissue sections of mice exposed to METH. Scale bar, 25 μm. **K** Quantification of the ratio of vascular medial thickness to total vessel size in the pulmonary artery from control and METH-exposed mice (*n* = 3). Two-tailed unpaired *t* test. **L** Representative immunofluorescent staining of nuclei (DAPI, blue) and α-SMA (red) in the lung tissues from control and METH-exposed mice. Scale bar, 25 μm. **M** Statistical analysis of the number of SMA^+^ cells in the lung tissues from control and METH-exposed mice (*n* = 3). Wilcoxon rank sum test. **N** Representative images of Masson-stained lung tissue sections of mice exposed to METH. Scale bar, 25 μm. **O** Quantification of the fibrotic areas (%) by normalizing Masson’s positive staining area to the total tissue area in the images analyzed in the pulmonary artery from the mice exposed to METH (*n* = 3). Two-tailed unpaired *t* test. **P** Photograph after pulmonary arteriography. The data are shown as the mean ± SD values. **P < 0.01, ***P < 0.001. CSA, cross-sectional area; WT, wild type

To determine whether METH abuse could induce PH-like pathological change in pulmonary arteries, we collected lung tissues from eight human cadavers (Table 1) who succumbed to METH abuse (METH abuse history, 1–7 years) and observed the pathological changes after H&E and Masson staining. Among the eight lung tissue samples, four had typical pathological changes of pulmonary hypertension, such as thickening of pulmonary arteriolar media, narrowing of the lumen, and vascular fibrosis (Fig. 1A–D), indicating that METH abuse could indeed induce the occurrence of PAH. To further evaluate the roles of METH on pulmonary arteries, we constructed a mouse model of PH by improving on previously reported methods (Chen et al. 2017; Wang et al. 2013). As follows: intraperitoneal injections of METH (10 mg/kg/time, twice/day) were given to the mouse for 5 days, 2 days apart, for 10 weeks (Fig. 1E), which also mimics, to some extent, the chronic pattern of using, withdrawing and relapsing from METH in abusers.

**Table 1:** Human specimens used in the present study

Group	Control-1	Control-2	Control-3	METH-PAH-1	METH-PAH-2	METH-PAH-3	METH-PAH-4	METH-PAH-5	METH-PAH-6	METH-PAH-7	METH-PAH-8
Cause of death	Traffic accident	Traffic accident	Sudden coronary death	Sudden cardiac death	Multiple organs failure	Acute poisoning	Acute poisoning	Acute poisoning	Sudden cardiac death	Sudden death	Traffic accident
Sex	Male	Male	Male	Male	Male	Female	Male	Female	Male	Male	Male
Age (year)	30	38	60	39	36	20	32	24	40	53	38
History of METH abuse (year)	0	0	0	4	Unknown	1	3	3	5	7	2
Pathological changes typical of pulmonary hypertension	No	No	No	No	Yes	No	Yes	No	Yes	Yes	No

To assess the PH mouse model using our modified methods as above, the right ventricular systolic pressure (RVSP), which indirectly reflects the pulmonary artery pressure, was measured after 10 weeks of exposure to METH. The results showed the mean RVSP of mice in the control group was 22 mmHg; however, the mean RVSP of mice in the METH-treated group was up to 28 mmHg, which met the diagnostic criteria of PH (mean RVSP ≥ 25 mmHg) (Fig. 1F, G). In addition, the ratio of pulmonary artery acceleration time to pulmonary artery ejection time (PAT/PET) and RV fractional area change (RVFAC) was also tested. Echocardiographic results showed that both PAT/PET (Fig. 1H) and RVFAC (Fig. 1I) in METH group were reduced compared with control group. Furthermore, we checked the changes in pulmonary artery morphometry by H&E staining (Fig. 1J, K), α-SMA immunofluorescent staining (Fig. 1L, M), and Masson staining (Fig. 1N, O) and observed that the pulmonary arteries of the METH-treated mice were consistent with the pathological changes in the pulmonary arteries of the human pulmonary arteries of the METH abusers, such as obvious thickening of pulmonary artery media, narrowing of lumen, and vascular fibrosis. In addition, angiography showed that the pulmonary vascular density of the METH-exposed group was significantly lower than that of the control group (Fig. 1P), consistent with the results observed in patients with METH abuse (Zamanian et al. 2018). These results indicated that METH administration could successfully induce PH.

### METH-exposed induced phenotypic transformation of PASMCs

**Fig. 2:**
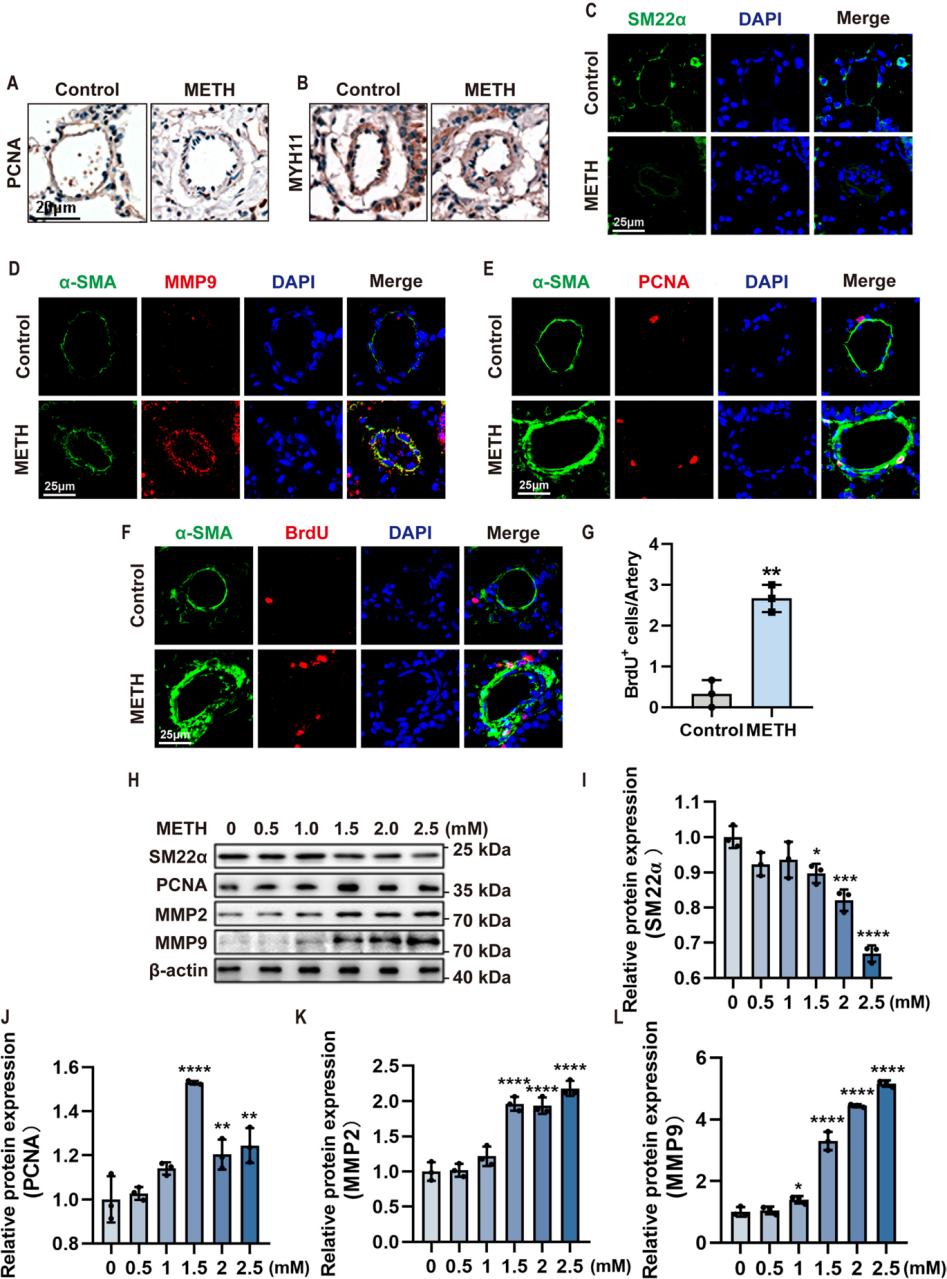
METH exposed induced phenotypic transformation of PASMCs. **A** Representative immunostaining of PCNA in lung tissues from control and METH-exposed mice. Scale bar, 20 μm. **B** Representative immunostaining of MYH11 in lung tissues from control and METH-exposed mice. Scale bar, 20 μm. **C** Representative immunofluorescent staining of nuclei (DAPI, blue) and SM22α (green) in lung tissues from control and METH-exposed mice. Scale bar, 25 μm. **D** Representative immunofluorescent staining of nuclei (DAPI, blue), MMP9 (red), and α-SMA (green) in lung tissues from control and METH-exposed mice. Scale bar, 25 μm. **E** Representative immunofluorescent staining of nuclei (DAPI, blue), PCNA (red), and α-SMA (green) in lung tissues from control and METH-exposed mice. Scale bar, 25 μm. **F** Representative immunofluorescent staining of nuclei (DAPI, blue), BrdU (red), and α-SMA (green) in lung tissues from control and METH-exposed mice. Scale bar, 25 μm. **G** Statistical analysis of the number of BrdU.^+^ cells in the lung tissues from control and METH-exposed mice (*n* = 3). Two-tailed unpaired *t* test. **H** Expression levels of phenotypic transformation-related proteins in cultured PASMCs, as determined by Western blotting. **I**–**L** Quantification of related protein levels shown in **F** after normalization to β-actin (*n* = 3). One-way ANOVA analysis with Bonferroni post hoc analysis. The data are shown as the mean ± SD values. *P < 0.05; **P < 0.01, ***P < 0.001, ****P < 0.0001

Previous studies demonstrated that PASMC phenotypic transformation played important roles in the pathological thickening of vascular media (Satoh et al. 2018; Ferguson et al. 2021; Diebold et al. 2010); thus, we investigated whether METH exposure could induce PASMC phenotypic transformation. Immunohistochemical staining results showed that the expression of PCNA (Fig. 2A), as a marker of proliferation phenotype, was increased in media of pulmonary arteries from METH-treated mice compared to control mice, while the expression of MYH11 (Fig. 2B), as a marker of contractile phenotype, was decreased in the media of pulmonary arteries from METH-treated mice compared to control mice. In addition, immunofluorescence detection of the mice’s lung tissues showed that the expression of contractile marker SM22α (Fig. 2C) was significantly reduced, while the expression of proliferative and migratory phenotypic markers MMP9 (Fig. 2D) and PCNA (Fig. 2E) was increased, which was accompanied by an increase in BrdU^+^ cells in PASMC (Fig. 2F, G). These were consistent with the previous immunohistochemical results. To verify the results observed in METH-exposed mice, the primary culture PASMCs was used to treat with METH to observe the change in related proteins. Western blot analysis showed that the expression of PCNA, MMP2, and MMP9 was increased, while the expression of SM22α was decreased in METH-treated PASMCs in a concentration-dependent manner (Fig. 2H–L) compared with control cells. These results indicated that METH exposure could induce PASMCs to switch from a contractile phenotype to a proliferative phenotype.

### Nupr1 deficiency alleviated METH-induced PH in mice

**Fig. 3:**
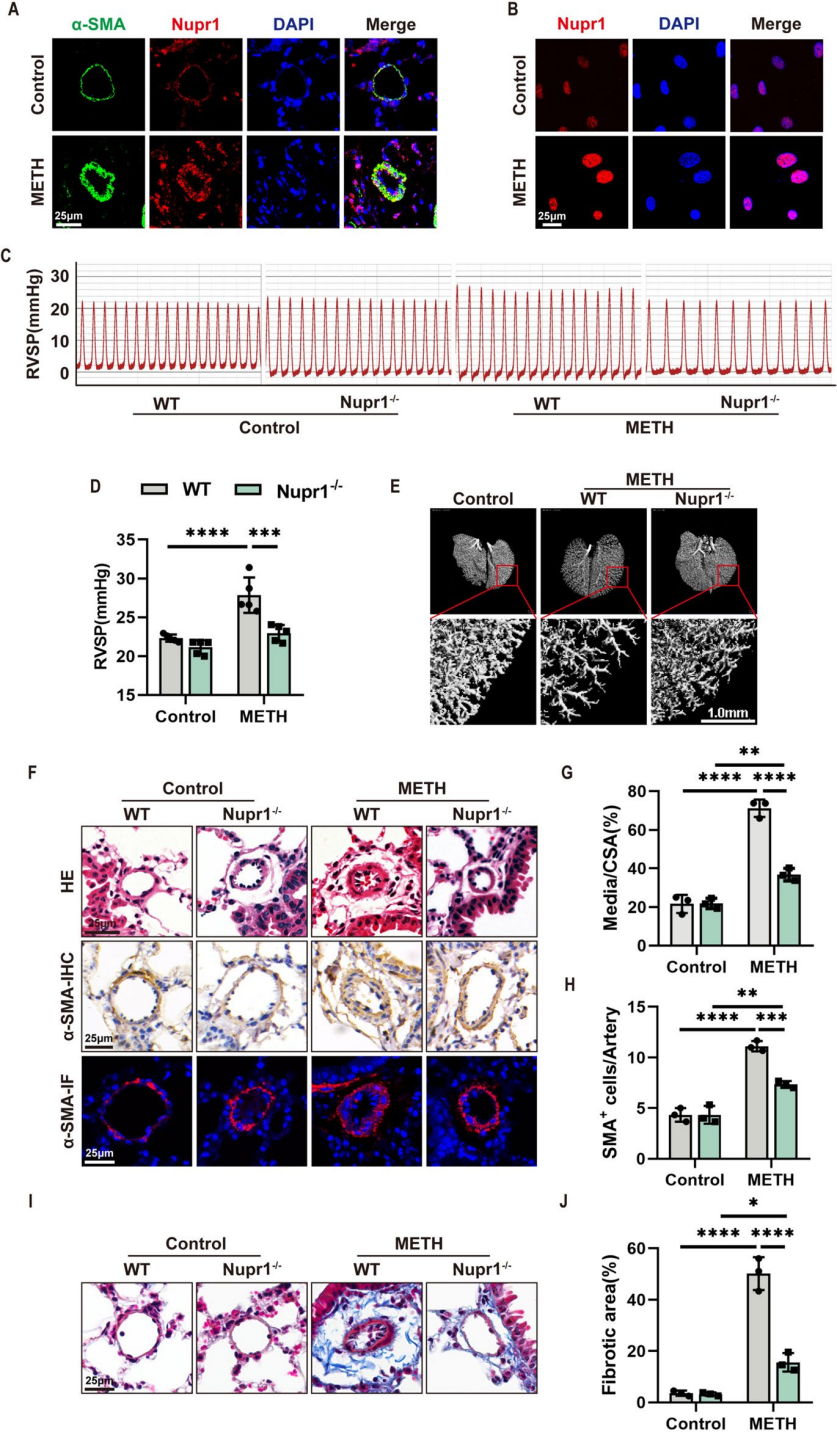
Nupr1 deficiency alleviated the METH-induced PH in mice. **A** Representative immunofluorescent staining of nuclei (DAPI, blue), Nupr1 (red), and α-SMA (green) expression in lung tissues from control and METH-exposed mice. Scale bar, 25 μm. **B** Representative immunofluorescent staining of Nupr1 (red) expression in cultured PASMCs treated with METH. Scale bar, 25 μm. **C** Pressure curve of right ventricular systolic pressure (RVSP) in WT and Nupr1^−/−^ mice with or without METH exposure. **D** Quantification analysis of RVSP in WT and Nupr1^−/−^ mice with or without METH exposure (*n* = 5). Two-way ANOVA with Bonferroni post hoc analysis. **E** CT scan after pulmonary angiography of WT and Nupr1^−/−^ mice with or without METH exposure. Scale bar, 1.0 mm. **F** Representative images of H&E staining, immunohistochemistry, and immunofluorescence of α-SMA in lung tissue sections of WT and Nupr1^−/−^ mice with or without METH exposure. Scale bars, 25 μm. **G** Quantification of the ratio of vascular medial thickness to total vessel size in the pulmonary arteries of WT and Nupr1^−/−^ mice with or without METH exposure (*n* = 3). Two-way ANOVA with Bonferroni post hoc analysis. **H** Statistical analysis of the number of SMA^+^ cells in the pulmonary arteries of WT and Nupr1^−/−^ mice with or without METH exposure (*n* = 3). Two-way ANOVA with Bonferroni post hoc analysis. **I** Representative images of Masson-stained lung tissue sections of WT and Nupr1^−/−^ mice with or without METH exposure. Scale bar, 25 μm. **J** Quantification of the fibrotic areas (%) after normalizing with Masson’s positive staining area to total tissue area in WT and Nupr1^−/−^ mice with or without METH exposure (*n* = 3). Two-way ANOVA with Bonferroni post hoc analysis. The data are shown as the mean ± SD values. **P < 0.01, ***P < 0.001, ****P < 0.0001. CSA, cross-sectional area; WT, wild type

Nuclear protein 1 (Nupr1 or P8) is a nuclear protein that regulates a variety of cellular events, including cell cycle, oxidative stress, apoptosis, autophagy, and DNA repair response (Cai et al. 2016; Zhou et al. 2021; Malicet et al. 2006; Ree et al. 1999; Vasseur et al. 1999; Yang et al. 2006). Previous studies demonstrated that METH exposure could induce Nupr1 expressions in cells, such as neurons (Xu et al. 2017) and endothelial cells (Cai et al. 2016), and elevated Nupr1 was reported to mediate METH-induced neuron and endothelial cell apoptosis. We hypothesized that Nupr1 might be involved in METH-induced PH. Therefore, we investigated whether exposure to METH altered Nupr1 expression. Our immunofluorescence results demonstrated that Nupr1 was significantly elevated in the pulmonary arterial media of mice exposed to METH (Fig. 3A). Furthermore, our double immunofluorescence staining showed colocalization of Nupr1 with α-SMA (Fig. 3A), which indicated that Nupr1 expression was located in the PASMCs of METH-exposed mice. Meanwhile, we also treated PASMCs with METH and detected Nupr1 expression by immunofluorescence staining. The results showed that METH significantly increased the expression of Nupr1 in PASMCs (Fig. 3B). These results provide reliable evidence that METH exposure could induce Nupr1 expression in PASMCs in vivo and in vitro.

**Fig. 4:**
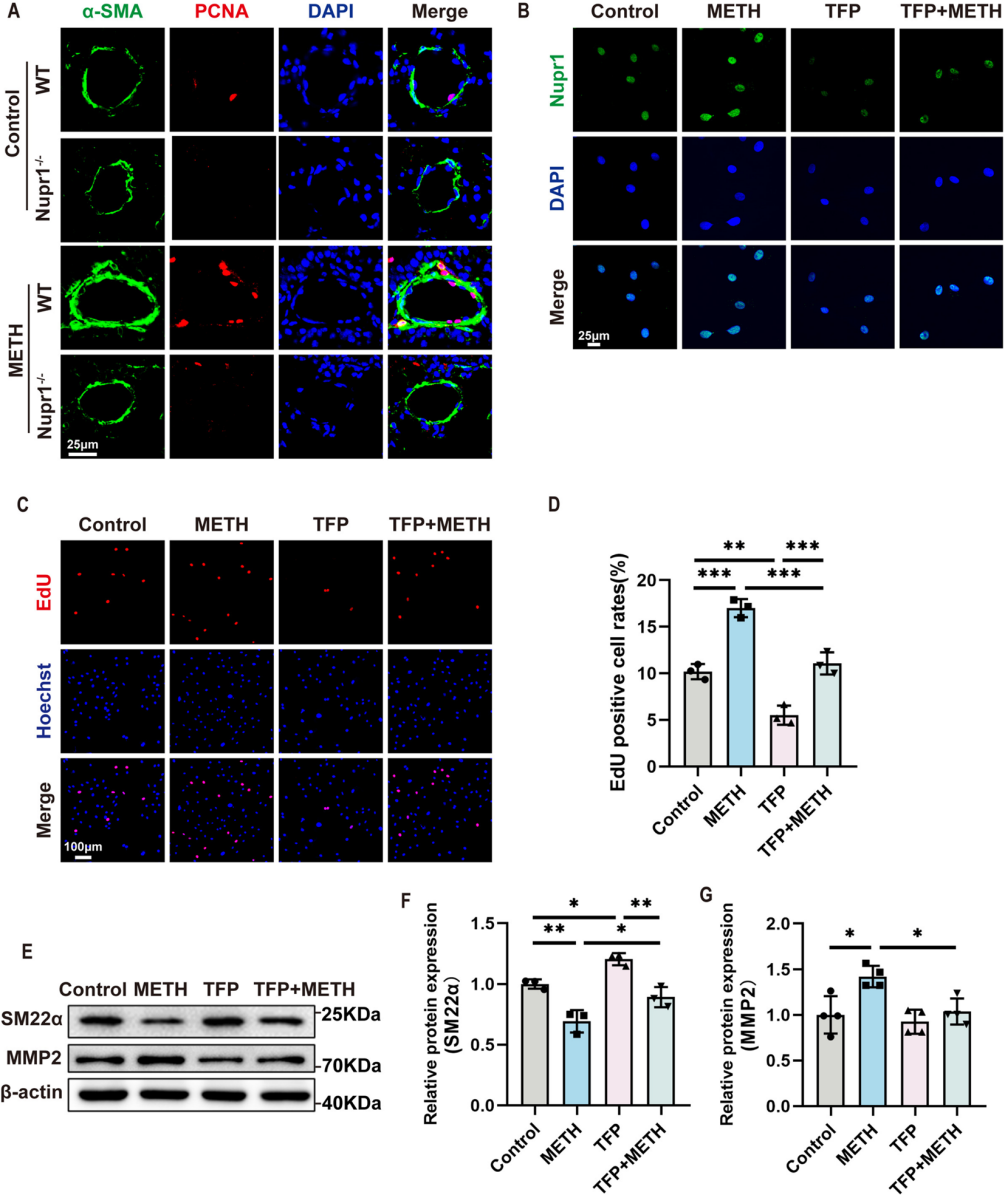
Nupr1 is involved in METH-induced PH via mediating PASMC phenotypic transformation. **A** Representative immunofluorescent staining of nuclei (DAPI, blue), PCNA (red), and α-SMA (green) expression in lung sections of METH-treated mice. Scale bar, 25 μm. **B** Representative immunofluorescent staining of nuclei (DAPI, blue) and Nupr1 (green) expression in cultured PASMCs. Scale bar, 25 μm. **C** Representative images of the EDU Cell Proliferation Assay. Scale bar, 100 μm. **D** Statistical analysis of the number of EdU-positive cells (*n* = 3). One-way ANOVA with Bonferroni post hoc analysis. **E** Expression levels of phenotypic transformation-related proteins in cultured PASMCs, as determined by Western blotting. **F**, **G** Quantification of related protein levels shown in D by normalized to β-actin (*n* = 3). One-way ANOVA with Bonferroni post hoc analysis. The data are shown as the mean ± SD values. *P < 0.05; **P < 0.01, ***P < 0.001

To ascertain the involvement of Nupr1 in METH-induced PH, we performed pulmonary hemodynamic and histological analyses in METH-treated wild-type (WT) and Nupr1-knockout mice (Nupr1^−/−^). We found that the METH-treated WT mice developed PH, with a significant increase in RVSP relative to WT control mice. However, Nupr1 deficiency alleviated METH-induced RVSP increment (Fig. 3C, D). Angiography and CT scans showed that Nupr1 deficiency reversed the loss of pulmonary small vessels (Fig. 4E). Nupr1 deficiency alleviated METH-induced pulmonary vascular remodeling in pulmonary arterials, as evidenced by decreased pulmonary vascular wall thickness (Fig. 4F–H) and vascular fibrosis (Fig. 4I, J). These data indicated that Nupr1 played important roles in METH-induced PH.

### Nupr1 was involved in METH-induced PH via mediating pulmonary arterial smooth muscle cell phenotypic transformation

Previous studies demonstrated that PASMC phenotypic transformation was a key step in the development of PH. To determine whether Nupr1 could mediate the pulmonary arterial smooth muscle cell phenotypic transformation, we observed the PCNA expression in Nupr1^−/−^ mice exposed to METH and found that Nupr1 deficiency could block the PCNA expression induced by METH exposed in the pulmonary arterial smooth muscle cell (Fig. 4A). These results indicated that Nupr1 deficiency could inhibit the phenotypic transition of pulmonary artery smooth muscle cells.

Trifluoperazine (TFP) is an antipsychotic drug reported to be an effective inhibitor of Nupr1 and have an antitumor role (Santofimia-Castano et al. 2019). Therefore, we used TFP as a specific inhibitor of Nupr1 to investigate the roles of Nupr1 in PASMC phenotypic transformation. Then, TFP-pretreated PASMCs were treated with METH, and the expression of Nupr1 was assessed through immunofluorescence staining. The results demonstrated an increase in Nupr1 expression in METH-treated PASMCs, and TFP could effectively inhibit the expression of Nupr1 induced by METH (Fig. 4B). At the same time, the proliferation of PASMCs was examined, revealing a significant increase in EdU-positive cell rates after METH treatment. In contrast, inhibiting Nupr1 expression with TFP led to a reduction in the EdU‐positive cell rates (Fig. 2C, D). Meanwhile, to evaluate the effects on PASMC phenotypic transformation by inhibiting Nupr1 expression, we detected the expression of SM22α and MMP2 in PASMCs by Western blot and found that SM22α expression was decreased, but MMP2 expression was increased after METH exposure, while these changes were reversed following the Nupr1 inhibition (Fig. 4E–G). These results suggested that inhibition of Nupr1 expression could attenuate PASMCs’ transition from contractile to proliferative.

### Nupr1 mediated METH-induced PH via the STIM1 pathway

**Fig. 5:**
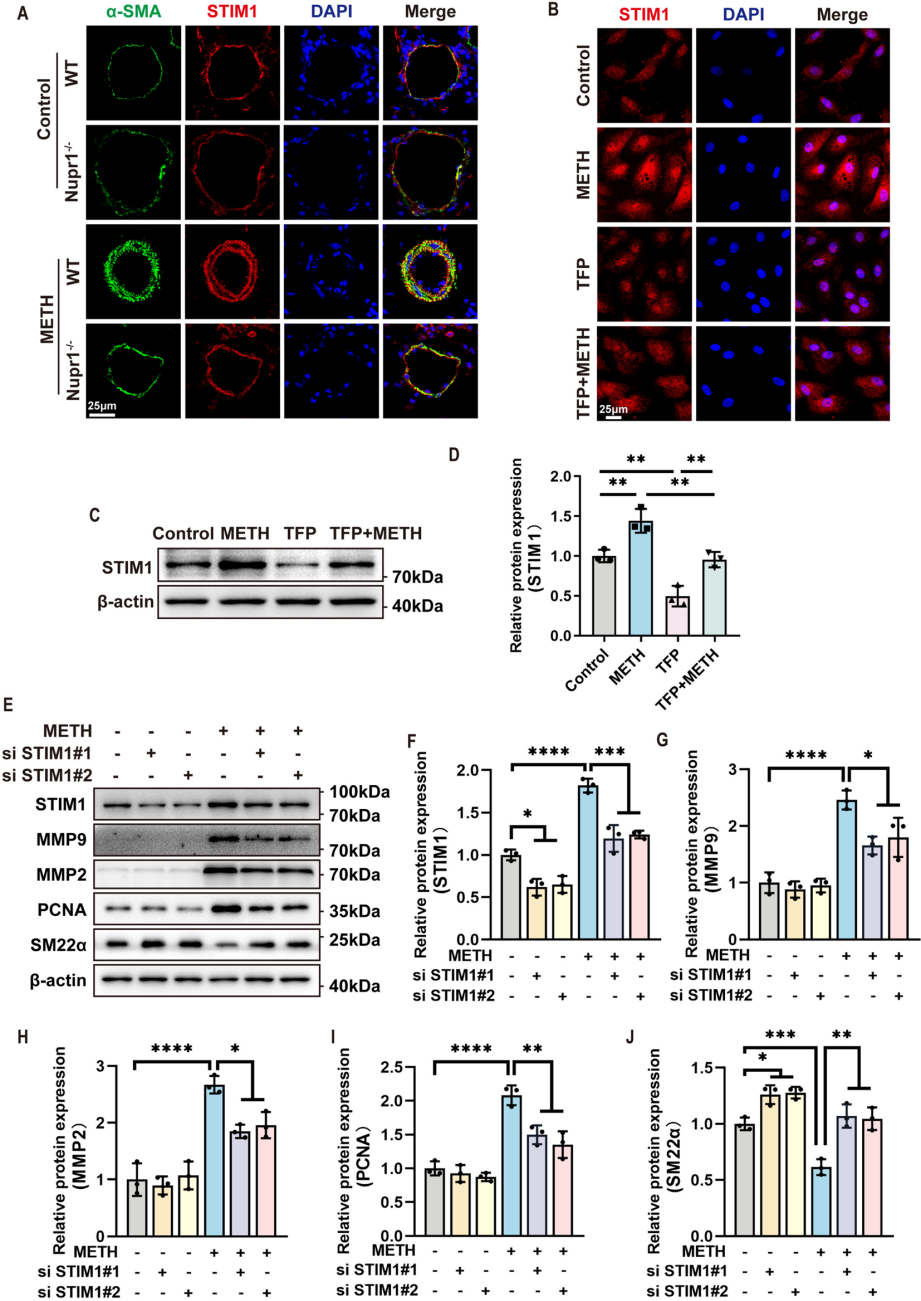
Nupr1 mediated METH-induced PH via the STIM1 pathway. **A** Representative immunofluorescent staining of nuclei (DAPI, blue), STIM1 (red), and α-SMA (green) expression in lung sections of WT and Nupr1.^−/−^ mice exposed to METH. Scale bar, 25 μm. **B** Representative immunofluorescent staining of nuclei (DAPI, blue), STIM1 (red) expression in PASMCs pretreated with TFP and exposed to METH. Scale bar, 25 μm. **C** Expression levels of STIM1 in PASMCs pretreated with TFP and exposed to METH, as determined by Western blotting. **D** Quantification of related protein levels shown in C after normalization to β-actin (*n* = 3). One-way ANOVA with Bonferroni post hoc analysis. **E** Expression levels of smooth muscle phenotypic transition associated proteins after silencing STIM1, as determined by Western blotting. **F**–**J** Quantification of related protein levels shown in **E** by normalized to β-actin (*n* = 3). One-way ANOVA with Bonferroni post hoc analysis. The data are shown as the mean ± SD values. *P < 0.05, **P < 0.01, ***P < 0.001, ****P < 0.0001

As previously mentioned, Nupr1 is involved in ER stress, which is associated with the Store-operated Ca^2+^ channels (SOCCs). STIM1, Orai1, and TRPC1 are recognized as molecular components of SOCCs. STIM1 in ER membranes serves as a sensor for ER Ca^2+^ level and mediates store-operated Ca^2+^ entry (SOCE) (Zhu et al. 2021). In order to establish whether STIM1 is implicated in Nupr1-mediated METH-induced PH, we first detected the change in STIM1 expression following Nupr1 blockade. We found that STIM1 expression was significantly increased in the pulmonary arteries from METH-treated mice and cultured PASMCs treated with METH compared with controls, and these changes could be reversed following Nupr1 inhibition (Fig. 5A–D). These data suggested that Nupr1 regulated STIM1 expression.

Given that STIM1 expression is regulated by Nupr1 and previous studies demonstrated that STIM1 is involved in PASMC phenotypic transformation (Ng et al. 2012; Wang et al. 2017; He et al. 2018). To determine whether STIM1 is also involved in METH-induced PASMC phenotypic transformation, we used siRNA to silence STIM1 expression in PASMCs and exposed PASMCs with METH, following which the expression of STIM1, PCNA, MMP2, MMP9, and SM22α were detected by Western Blot. The results revealed a substantial increase the expression of STIM1, PCNA, MMP2, and MMP9 were significantly increased, along with a significant decrease in SM22α expression in PASMCs treated with METH compared with control. Both siRNA#1 and siRNA#2 targeting STIM1 could effectively silence the expression of STIM1 and reverse the changes in PCNA, MMP2, MMP9, and SM22α expression induced by METH (Fig. 5E–J), indicating that STIM1 was involved in METH-induced PASMC phenotypic transformation. These data suggested that Nupr1 mediated METH-induced PH through the STIM1 pathway.

### STIM1 regulated PASMC phenotypic transformation by regulating Ca^2+^ channels

**Fig. 6:**
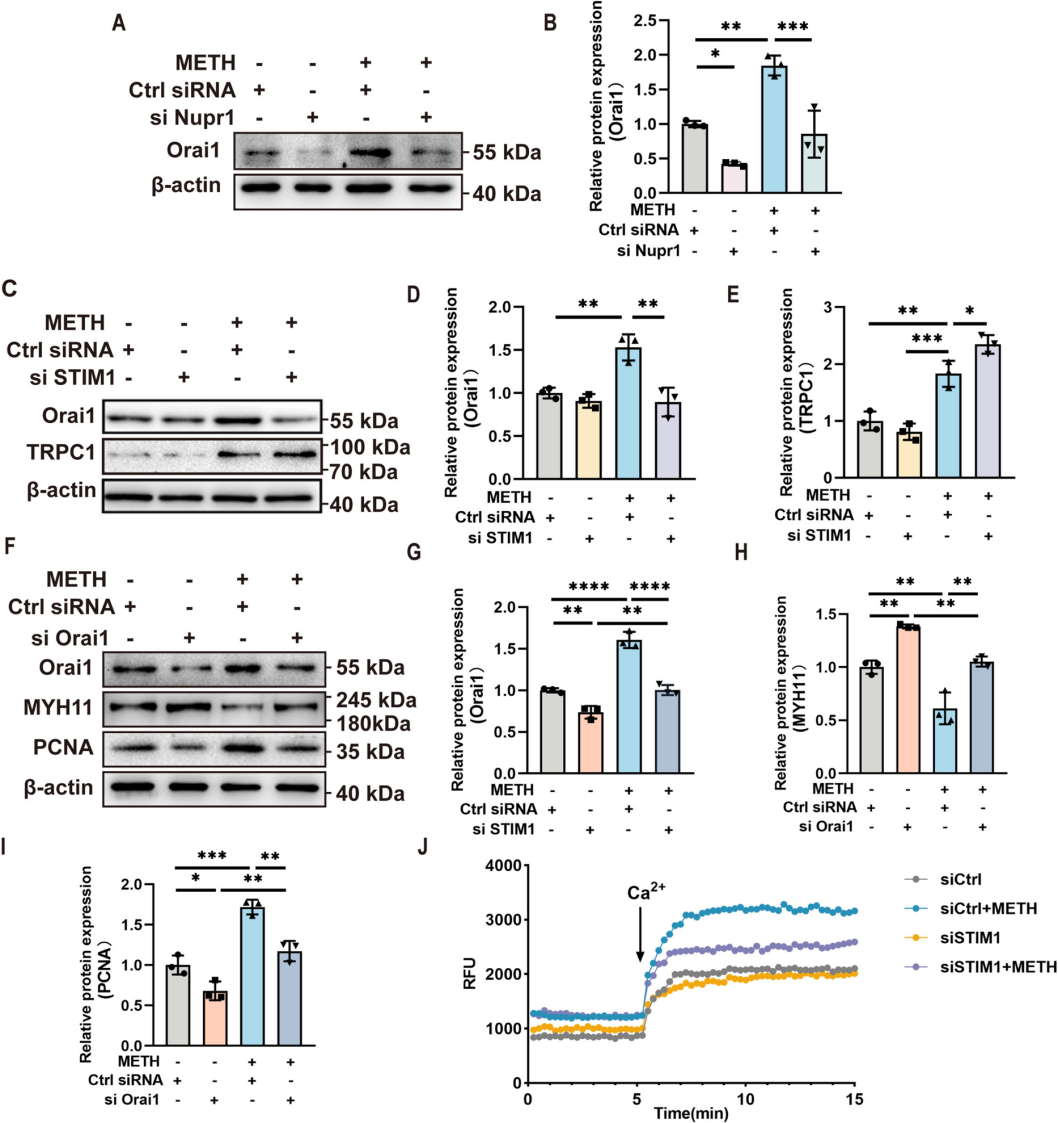
STIM1 regulated the phenotypic transformation of PASMCs by regulating Ca^2+^ channels. **A** Expression levels of Orai1 after silencing Nupr1, as determined by Western blotting. **B** Quantification of Orai1 levels shown in A after normalization to β-actin (*n* = 3). One-way ANOVA with Bonferroni post hoc analysis. **C** Expression levels of Orai1 and TRPC1 after silencing STIM1, as determined by Western blotting. **D** Quantification of Orai1 levels shown in **A** after normalization to β-actin (*n* = 3). One-way ANOVA with Bonferroni post hoc analysis. **E** Quantification of TRPC1 levels shown in **A** after normalization to β-actin (*n* = 3). One-way ANOVA with Bonferroni post hoc analysis. **F** Expression levels of Orai1, MYH11, and PCNA after silencing Orai1, as determined by Western blotting. **G**–**I** Quantification of related protein levels shown in **D** after normalization to β-actin (*n* = 3). One-way ANOVA with Bonferroni post hoc analysis. **J** The intracellular calcium contention curve of PASMCs exposed to METH after silencing STIM1. The data are shown as the mean ± SD values. **P < 0.01, ****P < 0.0001. RFU, relative fluorescence intensity

Since it has been shown that STIM1 is involved in METH-induced PASMC phenotypic transformation, the next question was how STIM1 regulated the phenotypic transformation of PASMCs. Considering that STIM1 is an important component of SOCE, we first detected the expression of Orai1 and TRPC1, the other two important components of SOCE. The results showed that the expression of Orai1 and TRPC1 was significantly increased after METH exposure, and after silencing STIM1, the elevated expression of Orai1 was inhibited while the expression of TRPC1 did not change (Fig. 6A–C), suggesting that STIM1 mediated PASMC phenotypic conversion induced by METH through the Orai1 pathway but not through the TRPC1 pathway. Next, we used siRNA targeting Orail to silence Orai1 expression in PASMCs and exposed them to METH. The expressions of Orai1, MYH11, and PCNA were detected by Western blot. The results showed that silencing the expression of Orai1 could reverse the changes in MYH11 and PCNA expression induced by METH (Fig. 6D–G), which further indicated that Orai1 was involved in STIM1-mediated phenotypic switching of PASMCs. In addition, we detected the change in Ca^2+^ content after METH treatment and found that the Ca^2+^ content was significantly increased compared with the control group, and silencing the expression of STIM1 inhibited the increase of Ca^2+^ content (Fig. 6H). These results suggested that STIM1 regulated the opening of SOCE through the Orai1 pathway to regulate Ca^2+^ influx and further regulated the phenotypic transition of PASMCs.

### Nupr1 expression was relevant to human METH-PAH

**Fig. 7:**
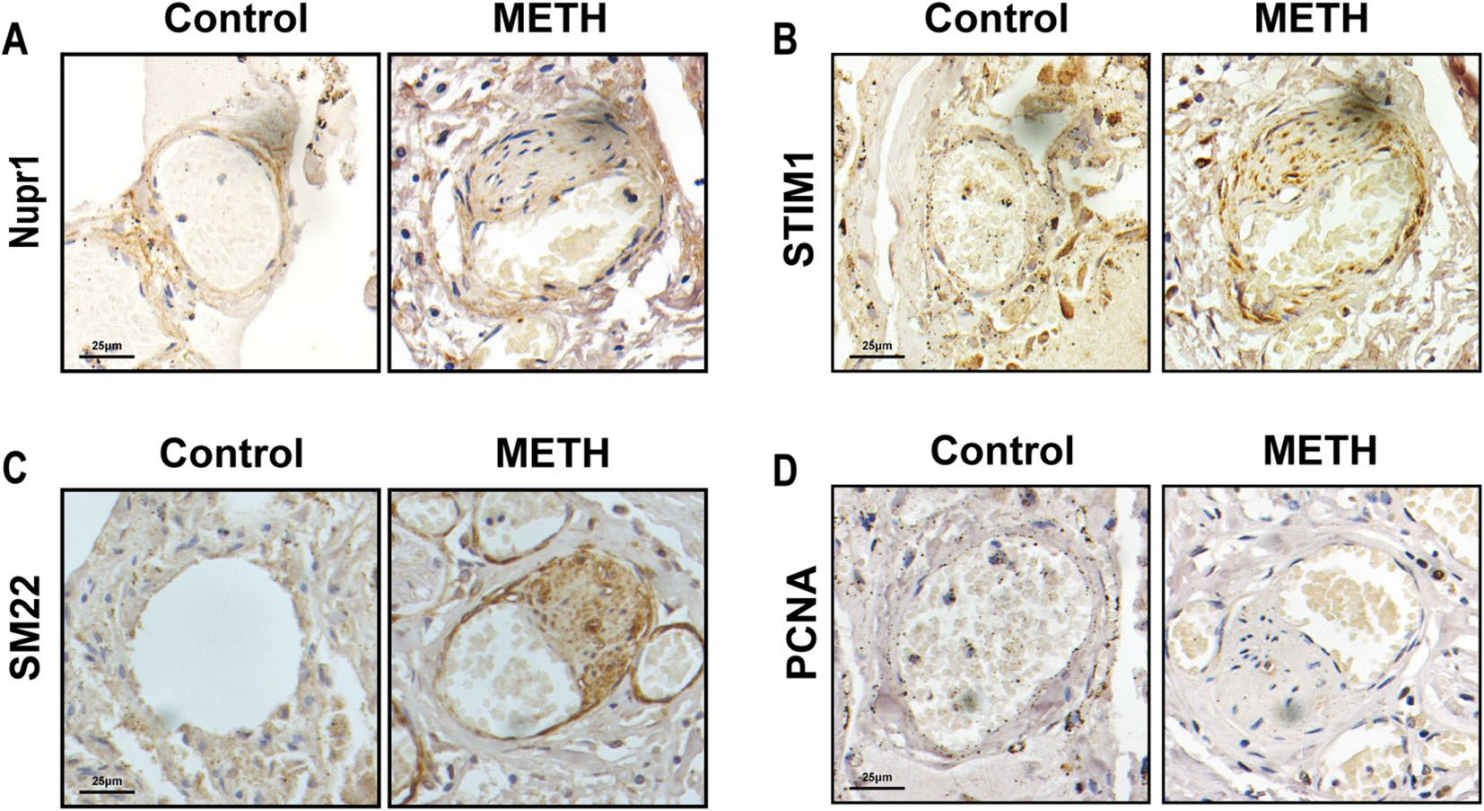
Nupr1 expression was relevant to human METH-PAH. **A** Immunohistochemical staining of Nupr1 of lung sections from deceased individuals with a METH abuse history. Scale bar, 25 μm. **B** Immunohistochemical staining of STIM1 of lung sections from deceased individuals with a METH abuse history. Scale bar, 25 μm. **C** Immunohistochemical staining of SM22α in the lung sections from deceased individuals with a METH abuse history. Scale bar, 25 μm. **D** Immunohistochemical staining of PCNA of lung sections from deceased individuals with a METH abuse history. Scale bar, 25 μm

To ascertain the potential implication of Nupr1 in human METH-PAH, we tested lung tissues from three specimens with a METH abuse history. Immunohistochemical staining of these human specimens distinctly illustrated a significant elevation in Nupr1 expression within the pulmonary arteries of individuals with a history of METH abuse, compared with normal pulmonary arteries (Fig. 7A). The same results were observed in STIM1 immunohistochemical staining experiments (Fig. 7B). In addition, we observed that the expression of SM22α in human pulmonary arteries from METH abusers was positive in the thickened portions of the pulmonary arteries, which indicated that these cells were characterized by SMCs (Fig. 7C). We also observed PCNA expression in their pulmonary arteries and found an increase in PCNA expression in the METH abusers compared with normal pulmonary arteries (Fig. 7D). Importantly, Nupr1, STIM1, and PCNA were presented in SM22α-positive cells. These results were consistent with those from METH-treated mice and cultured PASMCs treated with METH, confirming that Nupr1 indeed mediated the increase of STIM1 expression and induced the phenotypic transformation of PASMCs, which might be the underlying mechanism of human METH-related PAH.

## Discussion

**Fig. 8:**
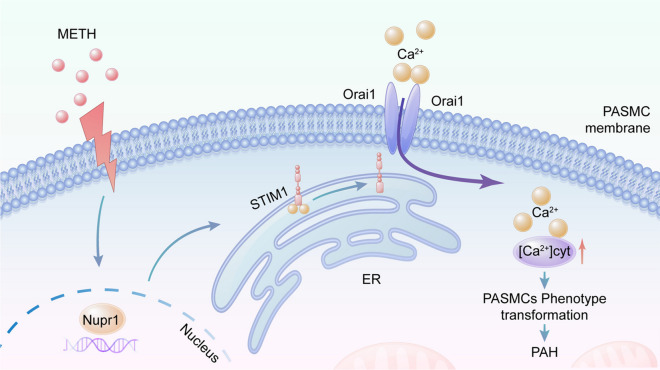
Mechanism of Nupr1 in the development of PH. When PASMCs were exposed to METH, the expression of Nupr1 in the cells increased, which led STIM1 on the ER to move to the cell membrane and bind with Orai1 on the cell membrane, causing the opening of Ca^2+^channels, an influx of extracellular Ca^2+^into the cells, and an increase in (Ca^2+^)_cyt_ in cells. The increased Ca^2+^ concentration led to the phenotypic transformation of PASMC and the formation of PH

PH is an extremely severe disease that can lead to fatalities. Currently, there are many causes of PH (Rabinovitch 2012; Kuhr et al. 2012), among which METH-PH has attracted increasing attention. METH is widely used in many countries and is a major cause of drug abuse (Zhao et al. 2018). Studies have shown that METH use can lead to PH (Zhao et al. 2018; Ramirez et al. 2018). However, the pathogenic mechanism of METH causing PH remained unclear. In this study, we successfully constructed a mouse model of METH-PH and found that METH induced phenotypic change in PASMCs. In addition, we found that Nupr1 expression was upregulated in the pulmonary arteries of the PH mouse models and human lung tissue samples exposed to METH. Nupr1 deficiency attenuated METH-induced PH and attenuated pulmonary vascular remodeling by inhibiting Ca^2+^ channel opening. And we found that Nupr1 activation promoted Ca^2+^ channel opening by promoting STIM1 expression. Thus, we concluded that Nupr1 activation promoted METH-induced PH through STIM1-mediated Ca^2+^ channel opening (Fig. 8).

Methamphetamine-associated pulmonary arterial hypertension (Meth-APAH) was found to be more common in men in previous studies (Ramirez et al. 2018). As reported in many previous studies (Ghazvini et al. 2016, 2021; Miller et al. 1998), estrogen has a protective effect on METH-induced neurotoxicity. In a study of the influence of estrogen on METH-induced neurotoxicity in mice, it was found that estrogen could reduce the toxic effects of METH on the nervous system by both inhibiting the ability of the dopamine transporter function and by lowering body temperature (Dluzen et al. 2002). Therefore, in order to avoid the effect of estrogen on our modeling, we chose male mice to construct the METH-PH mouse model. Two methods have been reported in the literature to establish METH-induced PH animal models. One method is the combination of METH with hypoxia to induce the occurrence of PH in mice (Kuhr et al. 2012), and the other is to use METH to continuously treat rats to induce PH (Chen et al. 2017). Both approaches have their advantages and disadvantages. In this present study, we combined and modified the above two methods and successfully established a new mouse METH-PH model to explore METH-induced PH and its mechanisms. Our proposed model simulated the long-term patterns of methamphetamine abusers’ use, withdrawal, and relapse. Our findings unequivocally demonstrated that METH abuse constituted a significant risk factor for the development of PH. Thus, the mouse model used in this present study could be helpful to further study the mechanism of METH-PH.

Vascular remodeling is an important pathological feature of PH that can lead to increased pulmonary vascular resistance and significant proliferation of PASMCs (Sakao et al. 2010). Transcription regulator Nupr1 is a cellular stress protein. Different external pressures and cellular microenvironments can affect its expression and mediate its entry into the nucleus to regulate the expression of various genes. Nupr1 can regulate the transcription of different genes in different intracellular and extracellular environments and participate in the occurrence and development of many diseases. Studies have shown that Nupr1 plays an upstream role in ER stress response during tetracycline-induced apoptosis in mouse and human tumor cells (Carracedo et al. 2006). Recent studies from our laboratory showed that the role of Nupr1 was also upstream of the endoplasmic reticulum stress response in the autophagy and apoptosis of nerve cells (Cai et al. 2016) and vascular endothelial cells (Xu et al. 2017) induced by METH. In addition, a study on the pathogenesis of PH highlighted the involvement of ER stress (Pan et al. 2020). Our study revealed the upregulation of Nupr1 expression in METH-PH. Nupr1 deficiency inhibited METH-induced PH and pulmonary vascular remodeling in mice. It has been demonstrated in previous experiments by our group that Nupr1 can be transcriptionally activated in response to oxidative stress or ER stress (Cai et al. 2016). Therefore, we did not further explore the mechanism of METH’s effect on Nupr1 in this study.

Studies have corroborated the essential role of STIM1 proteins in orchestrating the signaling cascade that connects Ca^2+^ storage depletion with the initiation of Ca^2+^ influx (Liou et al. 2005). STIM1 molecule in ER and Orai1 protein in plasma membranes are two major components of SOCE, which can bind to pore-forming complexes to regulate intracellular Ca^2+^ levels. Stimulation of STIM1 activates SOCE, resulting in sustained extracellular calcium influx (Liou et al. 2005). We detected that elevated Nupr1 could induce the upregulation of STIM1 expression, thereby mediating the phenotypic switch of PASMCs. STIM1 is one of the main components of SOCE. Silencing STIM1 can inhibit the opening of Ca^2+^ channels, thereby reversing the transition from contractile to proliferative PASMCs.

These results demonstrate that Nupr1 activation promotes Ca^2+^ influx through STIM1-mediated SOCE opening, thereby promoting METH-induced pulmonary vascular remodeling and PH. In this study, we not only explored the mechanism at animal in vivo and cellular levels but also validated the findings in human tissues, and the results showed that the unveiled mechanism could also be applicable in human tissues.

Interestingly, METH was administered for 10 weeks and allowed withdrawal for 4 weeks, and the RVSP was still up to 25 mmHg, reaching the PH diagnostic criteria (Supplementary Fig. 1). These indicated that the damage caused by METH to pulmonary arteries could be irreversible and might not be restored even after METH withdrawal, suggesting that the risk factors might have existed for a long time. Therefore, METH addicts still need regular check-ups after drug withdrawal. In addition, after silencing Nupr1 with siRNA, the increase of p62 and LC3Π in PASMCs induced by METH could be reversed, indicating that METH may inhibit autophagy in PASMCs, and Nupr1 may participate in this process (Supplementary Fig. 2).

Since this study showed that TFP inhibited phenotypic switching in smooth muscle cells at the cellular level, it remains unclear whether TFP can be used as a therapeutic agent for PH. In addition, the limitation of this study was that although we showed Nupr1 activation could promote the occurrence of METH-induced PH via the opening of STIM1-mediated Ca^2+^ channels, the mechanism through which Nupr1 regulates the expression of STIM1 needs to be further clarified.

## Supplementary Information

Below is the link to the electronic supplementary material.

Supplementary file1

High resolution

Supplementary file2

High resolution

## Data Availability

The data and materials of the study can be obtained from the corresponding author upon request.
