# Supplementary figures and images for: Nupr1-mediated vascular smooth muscle cell phenotype transformation involved in methamphetamine induces pulmonary hypertension
Source: Cell Biol Toxicol. 2024 Feb 13;40(1):13. doi: 10.1007/s10565-024-09849-6 (PMC10861617; doi:10.1007/s10565-024-09849-6)

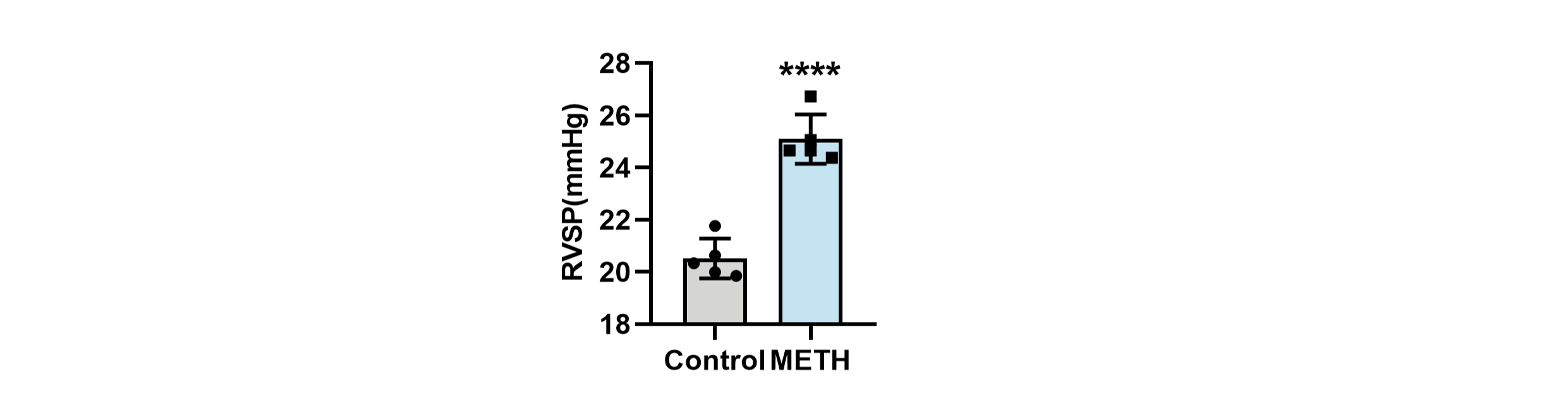

Supplement: Supplementary file 1 — Supplementary file1 [file 10565_2024_9849_Fig9_ESM.png]

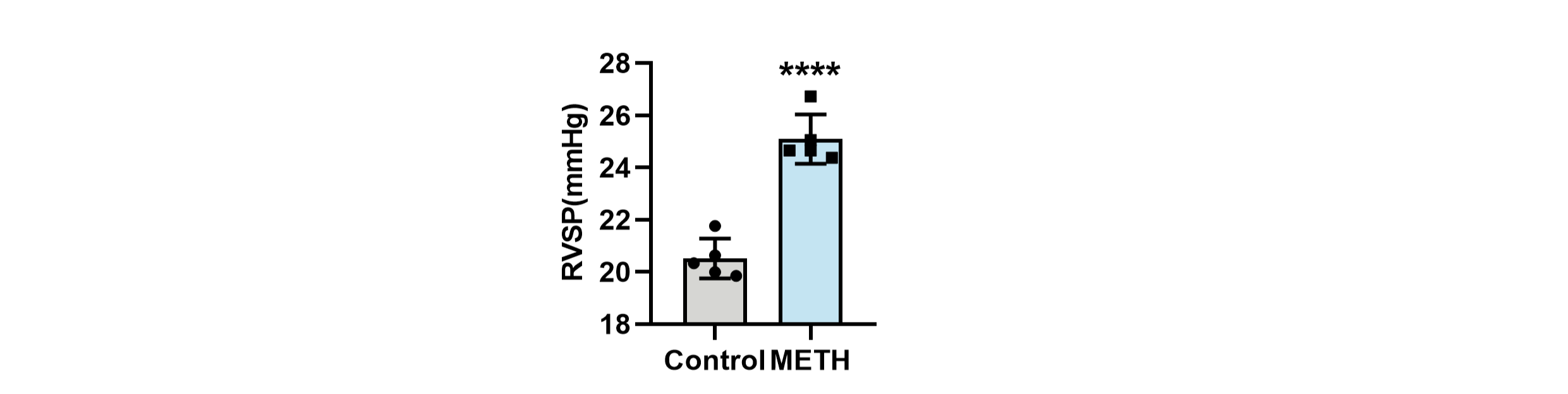

Supplement: Supplementary file 2 — High resolution [file 10565_2024_9849_MOESM1_ESM.tif]

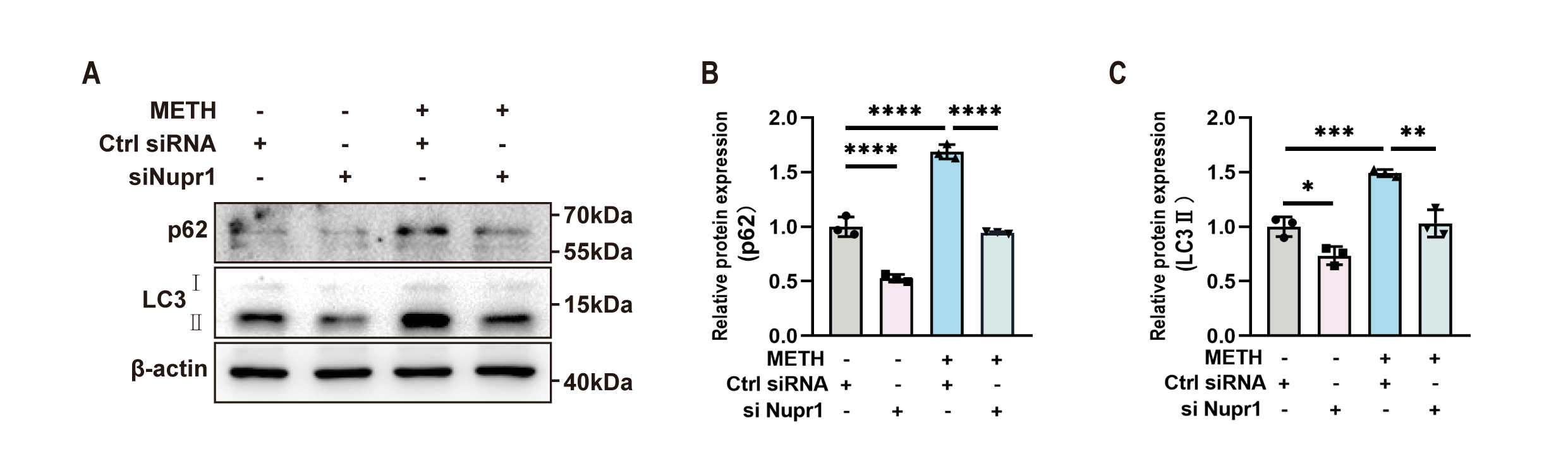

Supplement: Supplementary file 3 — Supplementary file2 [file 10565_2024_9849_Fig10_ESM.png]

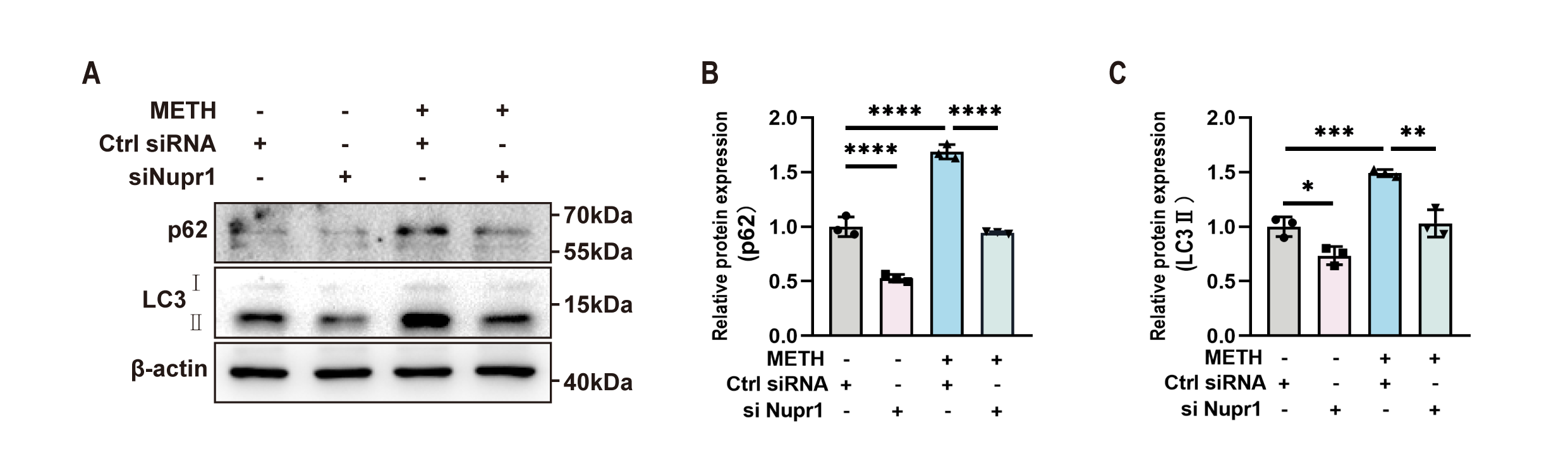

Supplement: Supplementary file 4 — High resolution [file 10565_2024_9849_MOESM2_ESM.tif]
